# A Flavonoid‐Rich Extract of *Scoparia dulcis* L. Exhibits Antiviral Activity against Herpes Virus Type 1

**DOI:** 10.1002/cbdv.202502903

**Published:** 2026-01-08

**Authors:** Francisco Leandro Medeiros de Lucena Jales, Emerson Michell da Silva Siqueira, Renato Dantas‐Medeiros, Edilane Rodrigues Dantas de Araújo, Jovelina Samara Ferreira Alves, Hugo Alexandre de Oliveira Rocha, Norberto Peporine Lopes, Leandro de Santis Ferreira, Emanuella de Aragão Tavares, Silvana Maria Zucolotto

**Affiliations:** ^1^ Department of Pharmacy Federal University of Rio Grande Do Norte (UFRN). Av. General Gustavo Cordeiro de Farias, Petrópolis Natal Brazil; ^2^ Department of Biochemistry Federal University of Rio Grande Do Norte (UFRN). Av. Salgado Filho 3000, Campus Universitário Lagoa Nova Natal Brazil; ^3^ Faculty of Pharmaceutical Sciences of Ribeirão Preto, Department of Biomolecular Sciences, Ribeirão Preto University of São Paulo São Paulo Brazil

**Keywords:** *Capraria dulcis*, antiviral agent, cytotoxicity, HSV‐1

## Abstract

This study investigated the phytochemical profile, cytotoxicity, and anti‐herpetic activity of the hydroethanolic extract from *Scoparia dulcis* L. aerial parts. Mass spectrometry revealed the presence of 15 compounds. The extract showed low cytotoxicity in Vero cells, maintaining over 80% viability at concentrations up to 250 µg/mL. In vitro antiviral assays demonstrated that the extract significantly inhibited HSV‐1 infectivity and increased cell protection at 25, 50, and 100 µg/mL. These results support the traditional medicinal use of *S. dulcis* and suggest its potential as a natural antiviral agent. The presence of multiple active compounds may contribute to its efficacy through synergistic effects. Overall, the findings encourage further investigation of *S. dulcis* as a source of bioactive molecules with potential for the development of alternative therapies against herpes simplex virus type 1.

## Graphical abstract

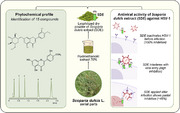

## 1 Introduction

*Scoparia dulcis* L., commonly known as sweet broom, is a medicinal plant belonging to the Plantaginaceae family. It is widely distributed across tropical and subtropical regions, including Brazil, India, the West Indies, and Myanmar [1, 2]. Although not native to Brazil, it is extensively used in traditional medicine and has attracted scientific interest due to its diverse pharmacological activities. Studies have demonstrated its antimicrobial [3], anti‐inflammatory [4, 5], antiviral [6], antiulcer [7], antitumor [8], and hypoglycemic properties [5,9]. These effects are associated with its complex phytochemical composition, including flavonoids, alkaloids, terpenoids, and other bioactive compounds [2, 10]. Among its reported therapeutic potentials, antiviral activity is particularly relevant in the context of increasing resistance to conventional drugs.

Herpes simplex viruses (HSV‐1 and HSV‐2) are double‐stranded DNA viruses belonging to the Herpesviridae family, capable of establishing latent infections in sensory nerve ganglia and periodically reactivating to cause recurrent lesions. Among them, HSV‐1 primarily infects epithelial cells of the orofacial region and establishes latency in sensory neurons, from which it can reactivate under conditions such as stress or immunosuppression [11]. During its replication cycle, HSV‐1 binds to specific receptors on the host cell surface, penetrates through membrane fusion, and releases its genome into the nucleus, where viral gene expression and replication occur in a tightly regulated sequence of immediate‐early, early, and late phases. The newly assembled virions are transported to the cell membrane and released, leading to the characteristic cytopathic effects observed in infected cultures [11, 12].

HSV‐1 remains the most prevalent of the human herpes simplex viruses. According to 2020 estimates, approximately 3.8 billion individuals aged 0–49 years were seropositive for HSV‐1, a prevalence that has remained relatively stable over the past decade [13]. When extrapolated to include individuals aged 50–99 years, the total number of people living with HSV‐1 worldwide is estimated to exceed 5 billion. Regionally, HSV‐1 prevalence is highest in Africa (approximately 85%) and lowest in the Americas (around 52%) [14].

A hallmark of herpesvirus infection is its lifelong latency and potential for reactivation, triggered by factors such as fever, ultraviolet exposure, psychological or physical stress, trauma, and immunosuppression [11, 15]. This reactivation often leads to recurrent outbreaks, impacting quality of life and increasing the risk of viral transmission. Current antiviral treatments, such as acyclovir, reduce symptom severity and viral shedding but do not eliminate the virus or prevent latency [11, 16]. Moreover, the emergence of drug‐resistant HSV strains has intensified the search for alternative therapeutic agents.

In Brazil, Imunomax is currently the only herbal medicine registered at the National Health Surveillance Agency (Anvisa) with a therapeutic indication for the treatment of herpes simplex. Its active pharmaceutical ingredient is the hydroalcoholic extract obtained from the bark of Uncaria tomentosa (Rubiaceae), commonly known as cat's claw. The product is available as a topical gel‐cream formulation, standardized to 50 mg per gram, equivalent to 0.037 mg of oxindole alkaloids calculated as mitraphylline.

This scenario highlights a clear gap between traditional knowledge and available therapeutic options, reinforcing the importance of exploring new plant‐based alternatives. The choice of *S. dulcis* was guided by its traditional use and previously reported antiviral diterpenes, while HSV‐1 was selected as a representative model for initial antiviral screening due to its global prevalence and well‐established in vitro infectivity assays.

Previous studies have demonstrated the antiviral activity of *S. dulcis* and its isolated compounds against other viruses. Diterpenoids such as scopadulic acid B and scopadulin have shown inhibitory effects on Epstein–Barr virus and HIV replication [6, 17, 18, 19, 20], while flavonoid glycosides and benzoxazinones isolated from this species have exhibited additional antiviral and anti‐inflammatory properties. In the present study, rather than focusing on isolated compounds, we aimed to evaluate the potential synergistic antiviral effect of the hydroethanolic extract, reflecting the traditional medicinal use of *S. dulcis* and the interactions among its multiple phytoconstituents. This holistic approach may enhance antiviral efficacy while reducing toxicity, in line with the pharmacological complexity characteristic of medicinal plants.

It is important to highlight that Brazil provides a year‐round abundance of *S. dulcis* aerial parts as raw material. Thus, the investigation of *S. dulcis* may not only validate its ethnopharmacological use but also contribute to the diversification of safe and effective therapeutic strategies against HSV‐1. In this context, the present study aimed to investigate the in vitro antiviral activity against HSV‐1 of hydroethanolic extract from the aerial parts of *S. dulcis* and phytochemical composition.

## 2 Results and Discussion

### 2.1 Phytochemical Profile of *S. dulcis* Extract

The *S. dulcis* extract was analyzed by liquid chromatography‐diode array detection‐tandem mass spectrometry (LC‐DAD–MS/MS) in negative mode. Compound annotation combined deprotonated molecular ions [M–H]^−^ and diagnostic MS^2^ product‐ion patterns for *O*‐ and *C*‐glycosyl flavones (e.g., neutral losses of 162/176/308 Da for hexose, glucuronide, and rutinoside). The spectra were compared with previously reported data [21, 22] and the MassBank database (http://www.massbank.jp/) to assist in compound identification.

The resulting chromatogram (Figure 1) revealed multiple peaks, and a total of 15 secondary metabolites were annotated within a retention time range of 20.1 to 64.3 min (Table 1). The set is dominated by phenolic acids and flavones occurring as *C*‐ and *O*‐glycosides, plus late‐eluting *O*‐methoxylated aglycones. The observed elution order, phenolic acid, followed by glycosylated flavones, and finally *O*‐methylated aglycones, matches expectations for a C18 stationary phase and polar gradients. A complete set of extracted ion chromatograms is given in (Figures S1–S15).

**FIGURE 1:**
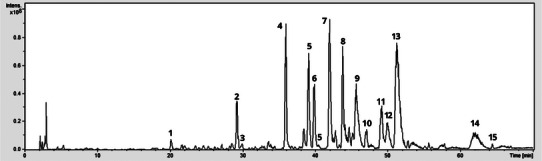
Liquid chromatography‐tandem mass spectrometry (LC‐MS/MS) chromatogram of *Scoparia dulcis* extract in negative mode.

**TABLE 1:** Data of liquid chromatography‐tandem mass spectrometry (LC‐MS/MS) of compounds characterized in *Scoparia dulcis* extract in negative mode.

Peak	Rt (min)	MS (*m/z*) [M‐H]^−^	MS^2^ (*m/z*)	Compound
1	20.1	342	179, 135	caffeoyl‐hexoside
2	28.5	417	285, 241, 152	kaempferol‐3‐*O*‐pentoside
3	29.9	431	269, 160, 117	apigenin‐*O*‐glucoside
4	35.9	593	575, 503, 473, 353, 383	vicenin‐2 (apigenin‐6,8‐di‐*C*‐glucoside)
5	39.0	563	545, 503, 473, 443, 383, 353	apigenin‐6‐*C‐*pentosyl‐8‐hexoside
6	39.8	563	545, 503, 473, 443, 413, 383, 353	apigenin‐6‐*C*‐hexosyl‐8‐pentoside
7	41.9	623	461, 443, 311	luteolin‐*O*‐hexoside^*^
8	43.7	769	607, 517, 441, 383	luteolin‐*O*‐hexosyl‐*O*‐glucuronide^*^
9	45.7	461	443, 369, 357	apigenin‐*C‐*pentoside
10	47.0	447	293, 149	p‐coumaroil^*^
11	49.2	607	299	diosmetin‐*O*‐rutinoside
12	50.0	461	299	diosmetin‐*O*‐hexoside
13	51.2	475	299, 175	diosmetin‐*O*‐glucuronide
14	62.1	299	269	diosmetin
15	64.3	327	291/229/171	trimethoxyflavone

^*^The compounds marked with an asterisk are putative: class is supported by MS/MS and DAD, but exact isomerism was not resolved.

The Peak 1 (20.1 min; m/z 342 [M–H]^−^) was assigned as caffeoyl‐hexoside (MS^2^ 179/135; loss of 162 Da to caffeate and subsequent CO_2_ loss). Peak 2 (28.5; m/z 417) was kaempferol‐3‐*O*‐pentoside (417→285/241/152; loss of 132 Da to the kaempferol aglycone). Peak 3 (29.9; m/z 431) was apigenin‐*O*‐glucoside (431→269/160/117; loss of 162 Da to apigenin). Peak 4 (35.9 min; m/z 593 [M–H]^−^) was assigned as vicenin‐2 (apigenin‐6,8‐di‐*C*‐glucoside) based on the diagnostic c‐glycoside cross‐ring fragments at m/z 575, 503, 473, 383, and 353, consistent with the flavone UV pattern (Band II ∼270 nm; Band I ∼335–340 nm).

Peaks 5–6 (39.0/39.8 min; m/z 563 [M–H]^−^) were assigned as apigenin‐6‐*C*‐pentosyl‐8‐*C*‐hexoside and apigenin‐6‐*C*‐hexosyl‐8‐*C*‐pentoside, supported by the diagnostic c‐glycoside cross‐ring product‐ion ladders at *m/z* 545, 503, 473, 443, 413, 383, and 353. Peak 7 (41.9; m/z 623) was a luteolin‐*O*‐hexoside (putative), dominated by 623→461/443/311 via loss of 162 Da. Peak 8 (43.7; m/z 769) was luteolin‐*O*‐hexosyl‐*O*‐glucuronide (putative), showing sequential losses of 162 and 176 Da (769→607/517/441/383). Peak 9 (45.7; m/z 461) was apigenin‐*C*‐pentoside, with abundant 18/92/104 Da cleavages typical of *C*‐glycosides. Peak 10 (47.0; m/z 447) was a minor p‐coumaroyl‐derived phenolic (447→293 with a prominent 149 fragment).

From peak 11 to peak 13, the diosmetin series was evident: peak 11 (49.2; m/z 607) diosmetin‐*O*‐rutinoside (607→299 by loss of 308 Da), peak 12 (50.0; m/z 461) diosmetin‐*O*‐hexoside (461→299 by loss of 162 Da), and peak 13 (51.2; m/z 475) diosmetin‐*O*‐glucuronide (475→299 and 175 by loss of 176 Da). At peak 14 (62.1 min; m/z 299 → 269, loss of 30 Da) and peak 15 (64.3 min; m/z 327 → 291/229/171), diosmetin and a trimethoxyflavone aglycone, respectively, showed small neutral losses, characteristic of *O*‐methylated flavones. Together with the earlier apigenin/luteolin *C*‐glycosides and their *O*‐glycosides, these signals delineate a phenolic profile dominated by flavone *C*‐glycosides, complemented by *O*‐glycosides and *O*‐methylated aglycones, in line with established UV/MS behavior for these classes.

The prominence of flavone *C*‐glycosides agrees with species‐level reviews of *S. dulcis*, which consistently report of presence of *C*‐glycosyl apigenins/luteolins as characteristic constituents in this species [2]. The detection of luteolin/apigenin *C*‐glycosides and *O*‐methylated aglycones (diosmetin/hispidulin‐type) is likewise consistent with prior chemical studies on *S. dulcis*. Notably, diosmetin itself has been reported in *S. dulcis* LC‐electrospray ionization/MS surveys, supporting the diosmetin series (peaks 11–14), and the observed diosmetin‐*O*‐glucuronide MS^2^ (m/z 475 → 299, 175) follows the glucuronide fragmentation described for diosmetin metabolites [21]. In addition, the minor *p*‐coumaroyl‐derived phenolic (peak 10) is coherent with prior isolations of *p*‐coumaroyl‐substituted flavonoid *O*‐glycosides from *S. dulcis* aerial parts extract [23].

The remarkable chemical diversity of metabolites identified in the *S. dulcis* extract is likely influenced by environmental factors such as intense solar exposure in Brazil's Northeast region and urban pollution. These environmental stressors have been widely cited in the literature as triggers of metabolic changes in plants. Other variables, including the time of collection, seasonal variations, and the choice of extraction solvent, may also have contributed to the observed chemical complexity [21, 24].

Previous phytochemical studies on *S. dulcis* have revealed a wide range of bioactive compounds beyond flavonoids. Yang et al. [10] employed 70% aqueous acetone for the extraction of aerial parts, followed by sequential partitioning into petroleum ether, ethyl acetate, and aqueous fractions. Through extensive chromatographic separation combined with spectroscopic analyses, they identified nine nitrogen‐containing metabolites, including benzoxazinone derivatives, several glucopyranosides, as well as 3,4‐dihydroxybenzeneacetic acid and zizyvoside I [10].

In another study, aqueous extracts obtained by macerating powdered leaves in distilled water were subjected to phytochemical screening and GC–MS analysis. This approach confirmed the presence of diverse classes of secondary metabolites, such as alkaloids, terpenoids, flavonoids, and steroids, highlighting the chemical diversity of *S. dulcis* when extracted with green solvents [25].

More recently, the ethyl acetate fraction of *S. dulcis* afforded the isolation of a novel diterpenoid, 2α‐hydroxyscopadiol. Structural characterization was achieved by chromatographic fractionation and spectroscopic techniques, and the compound demonstrated significant cytotoxic activity against breast cancer cell lines [26].

It is noteworthy that most previous phytochemical studies on *S. dulcis* employed organic solvents such as chloroform, dichloromethane, petroleum ether, or ethyl acetate to isolate diterpenoids, triterpenes, and other lipophilic constituents. While effective for recovering nonpolar metabolites, these solvents are generally considered toxic and less compatible with sustainable pharmaceutical development. In contrast, the present study adopted a hydroethanolic system, an ecologically safer and pharmaceutically acceptable solvent, which likely favored the detection of polar metabolites such as flavone glycosides and phenolic acids. This methodological choice is important not only because it aligns with green chemistry principles but also because it increases the translational potential of the extract, making it more suitable for future therapeutic applications and regulatory approval in phytomedicine development [27].

Future studies will include bioactivity‐guided fractionation to identify which compounds contribute most significantly to the antiviral activity of *S. dulcis*.

### 2.2 Cytotoxicity Assessment of *S. dulcis* Extract

**FIGURE 2:**
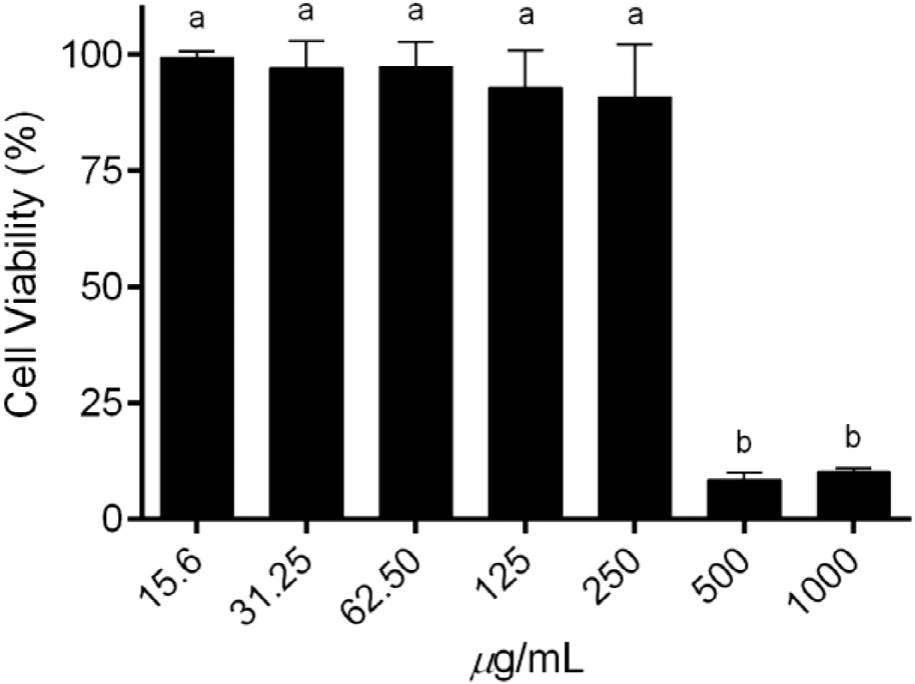
Cytotoxicity of *Scoparia dulcis* extract on Vero cells, evaluated using the MTT assay. Cell viability is expressed as a percentage relative to the untreated control (100%). Data were analyzed using one‐way analysis of variance (ANOVA) followed by Tukey's post hoc test. Different letters indicate statistically significant differences (*p* ≤ 0.0001).

The cytotoxic potential of *S. dulcis* extract was evaluated in Vero cells using the MTT assay across a concentration range of 15.6–1000 µg/mL, as shown in Figure 2. Cell viability remained above 85% at concentrations up to 250 µg/mL, with no statistically significant differences compared to the untreated control (p > 0.05). These results indicate that the extract is well tolerated within this concentration range, confirming its low cytotoxicity. In contrast, a marked and statistically significant reduction in cell viability was observed at higher concentrations (500 and 1000 µg/mL, *p* ≤ 0.0001), suggesting a dose‐dependent cytotoxic effect. Based on these findings, concentrations ≤250 µg/mL were considered non‐cytotoxic and were therefore selected for subsequent antiviral assays.

### 2.3 Antiviral Activity of *S. dulcis* Extract

**FIGURE 3:**
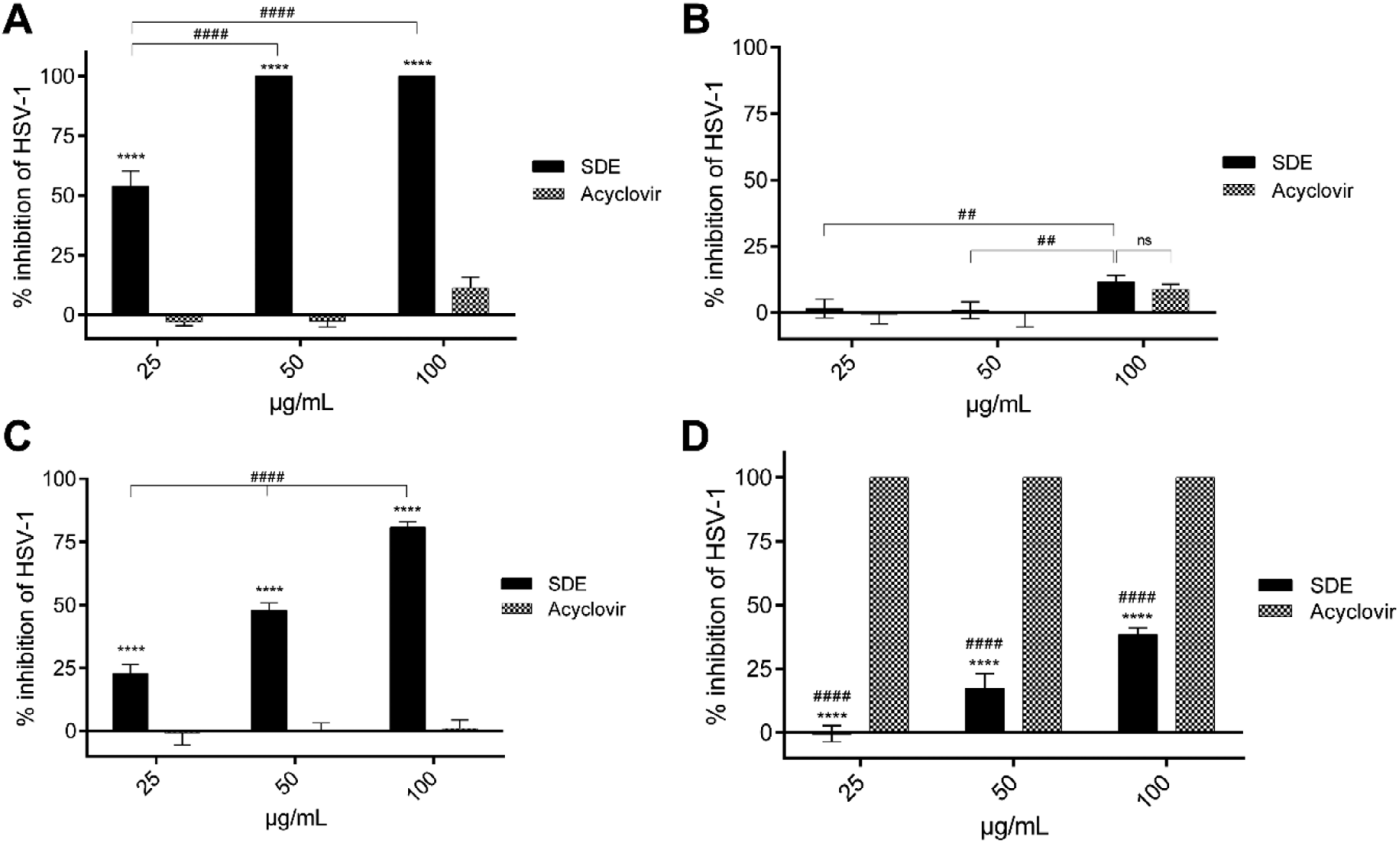
Antiviral effect of *Scoparia dulcis* extract against HSV‐1. Vero cells were treated with *Scoparia dulcis* extract (SDE) (25, 50, 100 µg/mL) or acyclovir (10^−2^ M) in four models: (A) direct inactivation, (B) pre‐treatment, (C) simultaneous treatment, and (D) post‐infection. Results represent mean ± SD (*n* = 4). Statistical analysis was performed using two‐way analysis of variance (ANOVA) with Tukey's post hoc test. ****p < 0.0001, SDE versus acyclovir at the same concentration; ## *p* < 0.01, #### *p* < 0.0001 versus SDE concentrations; ns = not significant.

To assess the antiviral potential of the extract, a direct activity assay was performed. The *S. dulcis* extract exhibited 100% inhibition of HSV‐1 infectivity at all tested concentrations (*p* < 0.0001), whereas acyclovir showed minimal activity even at 100 µg/mL (Figure 3A).

In the pre‐treatment model, where cells were incubated with the extract before infection, *S. dulcis* extract did not provide significant protection at lower concentrations (25–50 µg/mL). At 100 µg/mL, some activity was observed but remained significantly lower than acyclovir (*p* < 0.05) (Figure 3B). These findings suggest that the extract does not enhance host cell resistance to infection but rather acts directly on viral particles.

In the simultaneous treatment assay, where extract and viral inoculum were added together, *S. dulcis* extract exhibited protective activity at all tested concentrations (25–100 µg/mL). Interestingly, acyclovir did not demonstrate protective effects under these conditions (Figure 3C), suggesting that the extract may exert virucidal activity during the early stages of viral attachment or entry.

In the post‐infection model, Vero cells infected with HSV‐1 were subsequently treated with the *S. dulcis* extract (25–100 µg/mL). At the highest concentration, the extract inhibited viral replication by 48%, whereas acyclovir promoted nearly complete inhibition at all tested concentrations (*p* < 0.0001) (Figure 3D). The partial activity of the extract in this model suggests that its main mechanism may not involve interference with intracellular viral replication, as observed with acyclovir [28, 29].

Although no reports of antiviral activity against HSV‐1 have been identified for other *Scoparia* species, the activity observed for *S. dulcis* is consistent with findings in flavonoid‐rich medicinal plants. In particular, *C*‐ and *O*‐glycosylated flavonoids such as vitexin and isovitexin, commonly found in *Vitex* and *Passiflora* species, have been shown to inhibit HSV‐1 replication in vitro [30]. Likewise, mangiferin, a *C*‐glycosyl xanthone from *Mangifera indica*, demonstrated strong antiviral activity, including against acyclovir‐resistant HSV‐1 strains [31].

The pattern of activity observed in the direct contact and simultaneous treatment assays suggests that the hydroethanolic extract of *S. dulcis* acts mainly through a direct virucidal mechanism, possibly by interfering with viral adsorption or entry into host cells. Considering its complex phytochemical composition, this activity may result from a combination of bioactive metabolites acting synergistically at early stages of infection.

The antiviral effects observed may be related to the phytochemical profile of the *S. dulcis* extract. Flavonoids such as vitexin, isovitexin, and apigenin derivatives, identified in the extract, have been previously associated with anti‐HSV‐1 activity [32, 33]. Several studies have shown that flavonoids can act at different stages of the viral cycle, particularly by inhibiting viral entry and replication [34]. For instance, procyanidin, epicatechin, gallocatechin, and quercetin, flavonoids widely distributed in medicinal plants, have been shown to inhibit HSV‐1 replication in vitro [35, 36, 37, 38]. In addition, rutin, a glycosylated flavonoid, has demonstrated strong antiviral activity by interfering with viral enzymes and replication processes in other viruses, such as SARS‐CoV‐2 [36]. These findings support the hypothesis that glycosylated flavonoids may contribute to the virucidal activity observed for *S. dulcis*.

With regard to this species specifically, Hayashi [6] demonstrated that scopadulic acid B, a diterpene isolated from *S. dulcis*, exerts antiviral activity by directly inactivating viral particles and possibly interfering with early stages of infection, such as membrane fusion or DNA release. However, no direct evidence is currently available for the antiviral action of *S. dulcis* flavonoids against HSV, making the present findings particularly relevant. Future investigations could focus on elucidating the specific molecular targets involved, including viral envelope proteins and replication enzymes.

In addition to its direct virucidal effect, *S. dulcis* may modulate host immune responses. Previous studies have reported that flavonoids and diterpenoids from this species can influence cytokine production and macrophage activation, which may contribute indirectly to antiviral defense [19, 20, 39]. Further mechanistic studies are required to confirm whether such immunomodulatory pathways participate in the observed antiviral activity.

Taken together, these results highlight the potential of *S. dulcis* as a source of bioactive compounds with antiviral activity against HSV‐1. However, despite the promising in vitro results, it is essential to conduct in vivo studies to confirm both the antiviral efficacy and safety profile of the *S. dulcis* extract under physiological conditions. Such studies are crucial to evaluate pharmacokinetics, bioavailability, potential side effects, and therapeutic windows, which cannot be fully predicted through cell‐based models alone.

## 3 Conclusions

The *S. dulcis* extract, rich in flavonoids, exhibited low cytotoxicity (<250 µg/mL) and significant antiviral activity against HSV‐1, primarily through a direct virucidal mechanism. Although its post‐infection inhibitory effect was moderate compared to acyclovir, the extract completely suppressed viral infectivity in direct exposure assays.

Given its significant antiviral activity, the *S. dulcis* extract emerges as a promising candidate for the development of novel therapeutic agents, particularly against herpes simplex virus type 1 (HSV‐1). Nevertheless, additional in vivo investigations are necessary to validate its efficacy and to establish a comprehensive safety profile, thereby reinforcing its potential as a raw material for pharmacological formulations intended for human application.

## 4 Experimental

### 4.1 Material

HPLC‐grade acetonitrile and methanol were purchased from J T Baker, the 98%–100% formic acid was of analytical grade (Proquimius), and ethanol, n‐hexane, dichloromethane, ethyl acetate, and n‐butanol were of analytical grade (Quemis). Orientin (≥ 97%) and isoorientin (≥98%) reference standards used in TLC and LC‐MS comparative analysis were purchased from Sigma‐Aldrich. Water was purified with a Milli‐Q system (Millipore, Bedford, MA, USA). AlCl3 (Riedel‐de‐Haen) and NaOH (Synth) were used for flavonoid total content analysis. The mobile phases were filtered through a PVDF membrane (0.45 µm) (Merck). Vials (Analitica) and PVDF syringe filters (Vertical Chromatography) were used in preparing the samples for LC analysis.

### 4.2 Plant Material and Extract Preparation

The aerial parts of *S. dulcis* were collected in Natal, Rio Grande do Norte, Brazil, on March 15, 2018, between 8:00 and 9:30 a.m. (latitude: 5°43'57.0972'', longitude: 35°16'23.278''W), during the winter season as defined by the Agricultural Research Company of Rio Grande do Norte (EMPARN). Collection was authorized by the Biodiversity Authorization and Information System (SISBIO no. 35004) and the National Genetic Heritage Management System (SISGEN no. AC4F478). The plant specimen was identified by botanist Alan Roque de Araújo and deposited in the herbarium of Parque das Dunas, Natal, under registration number RN 1094. After collection, the plant material was dried in a circulating air oven at a temperature below 40°C for three days, then ground using an industrial blender. The hydroethanolic extract was prepared by maceration using ethanol: water (70:30, v/v) for 48 h. The solution was filtered, concentrated under reduced pressure using a rotary evaporator, and subsequently lyophilized (extraction yield of 11.4%).

### 4.3 Phytochemical Analysis by Mass Spectrometer

The chemical profile of the *S. dulcis* extract was evaluated by high‐performance liquid chromatography (HPLC) coupled with an ion trap mass spectrometer (amaZon SL, Bruker Daltonics, Billerica, USA). The chromatographic system consisted of an LC‐20AD solvent delivery unit, DGU‐20A3 degasser, CTO‐20A column oven, CBM‐20A controller, and SPD‐M20A diode array detector (200–400 nm) (Shimadzu, Kyoto, Japan). Samples were injected automatically (20 µL) using a SIL‐20A HT injector with a 100 µL loop. The extract was dissolved in methanol: water (1:1, v/v) at a concentration of 1 mg/mL and filtered through a PVDF syringe filter before analysis. Separations were performed on a Spherisorb ODS‐2 column (5 µm, 250 mm × 4.6 mm; Sigma‐Aldrich) at 25°C. The mobile phases were formic acid in ultra‐pure water 0.1% (solvent A) and formic acid in acetonitrile 0.1% (solvent B), at a flow rate of 1 mL/min under a gradient elution program: 5%–15% B (5–45 min), 15%–30% B (45–60 min), and 30%–100% B (60–70 min).

### 4.4 Cell Culture and Cytotoxicity Assay

Vero E6 cells (epithelial cell line derived from the kidney of *Chlorocebus aethiops*; RRID: CVCL_0574) were kindly provided by the Department of Internal Medicine, Faculty of Medicine of Ribeirão Preto, University of São Paulo (USP), Brazil. Cells were cultured in Dulbecco's Modified Eagle Medium (DMEM; Thermo Fisher), supplemented with 10% fetal bovine serum (FBS; Sigma‐Aldrich), and maintained at 37°C in a humidified atmosphere containing 5% CO_2_. The HSV‐1 viral stock (KOS strain, acyclovir‐sensitive) was obtained from the Faculty of Pharmacy, University of Rennes I, France. Viral propagation was performed in Vero cells, and titration was determined by plaque assay, expressed as plaque‐forming units per milliliter (PFU/mL), following the method described by Burleson et al. [40]. Viral aliquots were stored at −80°C until use.

The cytotoxicity of the *S. dulcis* extract and acyclovir was evaluated using the MTT assay, with minor modifications from the protocol described by Mosmann et al. [41]. Vero cells (2.0 × 10⁴ cells/well) were seeded in 96‐well plates and incubated for 24 h. Cells were then treated with serial concentrations of the extract (15.6, 31.25, 62.5, 125, 250, 500, and 1000 µg/mL) for 48 h. The test compounds were previously dissolved in 2% dimethyl sulfoxide (DMSO) and diluted in culture medium containing 2% FBS. Acyclovir was used as the reference compound under the same conditions. After incubation, the culture medium was removed, and 50 µL of MTT solution (1 mg/mL in phosphate‐buffered saline [PBS]) was added to each well and incubated for 4 h. Formazan crystals were dissolved by adding 100 µL of DMSO per well. Absorbance was measured at 540 nm using a microplate reader. Cell viability was expressed as a percentage relative to untreated control cells. The 50% cytotoxic concentration (CC_50_) was defined as the concentration that reduced cell viability by 50%.

### 4.5 Antiviral Assays

The antiviral assays described below were performed as described in previous articles [42, 43, 44] with minor modifications.

### 4.6 Direct Inactivation

The virucidal activity of the *S. dulcis* extract was evaluated based on its ability to inactivate HSV‐1 before cell infection, following the protocol described by Kesharwani et al. [43], with modifications. Briefly, extract at concentrations of 15.6, 31.25, 62.5, 125, 250, 500, and 1000 µg/mL, or acyclovir (50 µg/mL, positive control), were incubated with 100 PFU of HSV‐1 (titer: 3.75 × 10⁷ PFU/mL) in serum‐free DMEM for 60 minutes at 37°C. Following incubation, the mixtures were added to confluent Vero cell monolayers (2.0 × 10⁵ cells/well) and incubated for 1 h at 37°C to allow virus adsorption. After this period, supernatants were removed, cells were washed twice with PBS, and overlaid with DMEM containing 1% carboxymethylcellulose (CMC; Sigma‐Aldrich). After 48 h, cells were fixed and stained with naphthol blue black in 5% acetic acid, and viral plaques were counted. The 50% inhibitory concentration (IC_50_) was defined as the concentration required to reduce the number of plaques by 50% compared to untreated controls.

The full concentration range (15.6–1000 µg/mL) was used exclusively to determine the IC_50_ values. All subsequent mechanistic assays (pre‐treatment, simultaneous, and post‐infection) were conducted only with non‐cytotoxic concentrations (25, 50, and 100 µg/mL).

### 4.7 Pre‐treatment

For the pre‐treatment assay, Vero cells were seeded and cultured under the same conditions described for the virucidal test. Monolayers were pre‐treated with non‐toxic concentrations of the *S. dulcis* extract (25, 50, and 100 µg/mL) for 2 h at 37°C in a 5% CO_2_ atmosphere. After incubation, the extract was removed, and the cells were washed with PBS. HSV‐1 inoculum (100 PFU) was then added and allowed to adsorb for 2 h under the same conditions. Following viral adsorption, the medium was removed, cells were washed again, and 500 µL of overlay medium was added. Plates were incubated for 48 h at 37°C (5% CO_2_), then fixed, stained, and plaques were counted as described previously.

### 4.8 Simultaneous Treatment

The concomitant antiviral effect of the *S. dulcis* extract was evaluated as described by Sabouri Ghannad et al. [45], with adaptations. HSV‐1 (100 PFU) was mixed directly with different concentrations of the extract (25, 50, and 100 µg/mL) or acyclovir (11.1–44.4 µg/mL) in DMEM, and the mixture was immediately added to confluent Vero cell monolayers (2.0 × 10⁵ cells/well). The cells were incubated for 1 h at 37°C in a 5% CO_2_ atmosphere, with gentle agitation every 15 min to ensure uniform exposure. After incubation, the medium was removed, the cells were washed with PBS, and overlaid with DMEM containing 1% CMC. Plates were incubated for 48 h and processed as in previous assays.

### 4.9 Post‐infection

Confluent Vero cell monolayers were infected with HSV‐1 virus (100 PFU/well) for 1 h at 37°C. The virus was removed after its adsorption by washing with PBS, and the cells were covered with an overlay containing different concentrations of the extract (25, 50, and 100 µg/mL) and acyclovir (50 µg/mL). Plates were incubated for 48 h at 37°C and processed as previously described for the plaque reduction assay.

### 4.10 Statistical Analysis

Data from the antiviral assays were expressed as mean ± standard error of the mean (SEM) from two independent experiments performed in duplicate. The CC_50_ and IC_50_ values were calculated by non‐linear regression analysis of dose‐response curves using GraphPad Prism software version 8.0. Statistical comparisons between treatment groups (extracts or acyclovir) and the virus control (vehicle‐treated) were performed using one‐way analysis of variance, followed by appropriate post hoc tests when applicable. For the statistical analysis of acute oral toxicity, GraphPad Prism v8.0 software was used. Differences were considered statistically significant at *p* < 0.05.

## Author Contributions

Francisco Leandro Medeiros de Lucena Jales performed the experiments, analyzed the results, and wrote the manuscript. Emerson Michell da Silva Siqueira and Hugo Alexandre de Oliveira Rocha performed the in vitro experiments. Renato Dantas‐Medeiros and Edilane Rodrigues Dantas de Araújo writing—review & editing. Jovelina Samara Ferreira Alves, Leandro de Santis Ferreira, and Norberto Peporine Lopes performed the analysis by LC‐MS/MS. Emanuella de Aragão Tavares and Silvana Maria Zucolotto conceived and designed the experiments, analyzed the data, interpreted the results, and performed writing—review & editing of the manuscript. All authors read and approved the final manuscript.

## Conflicts of Interest

The authors declare no conflicts of interest.

## Supporting information




**Supporting File 1**: cbdv70744‐sup‐0001‐SuppMat.docx

## Data Availability

The publication contains all the data from this work. The plant species' use license is registered with SISGEN/Brazil ‐ no. AC4F478.
