# Supplementary material for: A Flavonoid‐Rich Extract of Scoparia dulcis L. Exhibits Antiviral Activity against Herpes Virus Type 1
Source: Chem Biodivers. 2026 Jan 8;23(1):e02903. doi: 10.1002/cbdv.202502903 (PMC12781047; doi:10.1002/cbdv.202502903)
Supplement: Supplementary file 1 — Supporting File 1: cbdv70744‐sup‐0001‐SuppMat.docx [file CBDV-23-e02903-s001.docx]

Suplemmentary Material

**Figure 1S.** ESI(–) CID–MS² of compound 1 (Rt 20.1 min; [M–H]− m/z 342). Product ions: 179, 135. Assignment: caffeoyl-hexoside.

**Figure 2S.** ESI(–) CID–MS² of compound 2 (Rt 28.5 min; [M–H]− m/z 417). Product ions: 285, 241, 152. Assignment: kaempferol-3-*O*-pentoside.

**Figure 3S.** ESI(–) CID–MS² of compound 3 (Rt 29.9 min; [M–H]− m/z 431). Product ions: 269, 160, 117. Assignment: apigenin-*O*-glucoside.

**Figure 4S.** ESI(–) CID–MS² of compound 4 (Rt 35.9 min; [M–H]− m/z 593). Product ions: 575, 503, 473, 383, 353. Assignment: vicenin-2 (apigenin-6,8-di-*C*-glucoside).

**Figure 5S.** ESI(–) CID–MS² of compound 5 (Rt 39.0 min; [M–H]− m/z 563). Product ions: 545, 503, 473, 443, 383, 353. Assignment: apigenin-6-*C-*pentosyl-8-*C*-hexoside.

**Figure 6S.** ESI(–) CID–MS² of compound 6 (Rt 39.8 min; [M–H]− m/z 563). Product ions: 545, 503, 473, 443, 413, 383, 353. Assignment: apigenin-6-*C*-hexosyl-8-*C*-pentoside.

**Figure 7S.** ESI(–) CID–MS² of compound 7 (Rt 41.9 min; [M–H]− m/z 623). Product ions: 461, 443, 311. Assignment: luteolin-*O*-hexoside (putative).

**Figure 8S.** ESI(–) CID–MS² of compound 8 (Rt 43.7 min; [M–H]− m/z 769). Product ions: 607, 517, 441, 383. Assignment: luteolin-*O*-hexosyl-*O*-glucuronide (putative).

**Figure 9S.** ESI(–) CID–MS² of compound 9 (Rt 45.7 min; [M–H]− m/z 461). Product ions: 443, 369, 357. Assignment: apigenin-*C*-pentoside.

**Figure 10S.** ESI(–) CID–MS² of compound 10 (Rt 47.0 min; [M–H]− m/z 447). Product ions: 293, 149. Assignment: p-coumaroyl-derived phenolic (tentative).

**Figure 11S.** ESI(–) CID–MS² of compound 11 (Rt 49.2 min; [M–H]− m/z 607). Product ion: 299. Assignment: diosmetin-*O*-rutinoside.

**Figure 12S.** ESI(–) CID–MS² of compound 12 (Rt 50.0 min; [M–H]− m/z 461). Product ion: 299. Assignment: diosmetin-*O*-hexoside.

**Figure 13S.** ESI(–) CID–MS² of compound 13 (Rt 51.2 min; [M–H]− m/z 475). Product ions: 299, 175. Assignment: diosmetin-*O*-glucuronide.

**Figure 14S.** ESI(–) CID–MS² of compound 14 (Rt 62.1 min; [M–H]− m/z 299). Product ion: 269. Assignment: diosmetin.

**Figure 15S.** ESI(–) CID–MS² of compound 15 (Rt 64.3 min; [M–H]− m/z 327). Product ions: 291, 229, 171. Assignment: trimethoxyflavone.
